# Comparative Performance of SARC-F, SARC-CalF, SARC-F + EBM, and Ishii Score for Detecting Sarcopenia in Hospitalised Geriatric Patients

**DOI:** 10.3390/jcm15072663

**Published:** 2026-04-01

**Authors:** Ioana Daniela Rus, Vlad Ionuț Nechita, Lucreția Avram, Dana Crișan, Cristina Pamfil, Laura Muntean, Elisabeta Ioana Hirișcău, Valer Donca

**Affiliations:** 1 Discipline of Geriatrics—Gerontology, Department 5—Medical Specialties, Faculty of Medicine, Iuliu Hațieganu University of Medicine and Pharmacy, 400012 Cluj-Napoca, Romania; valer.donca@umfcluj.ro; 2 Discipline of Medical Informatics, Research Methodology and Data Analysis, Department of Fundamental Sciences, Faculty of Nursing and Health Sciences (FAMSS), Iuliu Hațieganu University of Medicine and Pharmacy, 400012 Cluj-Napoca, Romania; nechita.vlad@umfcluj.ro; 3 Discipline of Internal Medicine, Department 4, Faculty of Medicine, Iuliu Hațieganu University of Medicine and Pharmacy, 400012 Cluj-Napoca, Romania; crisan.dana@umfcluj.ro; 4 Discipline of Rheumatology, Department 5—Medical Specialties, Faculty of Medicine, Iuliu Hațieganu University of Medicine and Pharmacy, 400012 Cluj-Napoca, Romania; cristinapamfil.umfcluj@gmail.com (C.P.); muntean.laura@umfcluj.ro (L.M.); 5 Nursing Department, Faculty of Nursing and Health Sciences (FAMSS), “Iuliu Hațieganu” University of Medicine and Pharmacy, 400012 Cluj-Napoca, Romania; ihiriscau@umfcluj.ro

**Keywords:** sarcopenia, SARC-F, SARC-CalF, Ishii score, EWGSOP2

## Abstract

**Background/Objectives:** Sarcopenia is a progressive decline in skeletal muscle strength and mass, leading to decreased functionality, metabolic disorders, morbidity, and mortality. There are a number of sarcopenia screening tools, such as the SARC-F questionnaire (that includes noting strength, assistance with walking, ability to raise from the chair, climb stairs, and falls), with its augmented forms that have added calf circumference (SARC-CalF), BMI and age (SARC-F + EBM), and the Ishii score, which show variable performance across populations. However, these were developed and validated mostly in Asian cohorts. To evaluate the diagnostic accuracy of these tools for the European Working Group on Sarcopenia in Older People (EWGSOP2), as well as define sarcopenia in hospitalized East European older adults, with sex and obesity stratification. **Methods:** Sarcopenia was diagnosed using the EWGSOP2. ROC analyses with DeLong tests assessed SARC-F, SARC-CalF, SARC-F + EBM, and the Ishii score in 278 Romanian inpatients (probable sarcopenia *n* = 201/278, 72.3%; confirmed *n* = 77/278, 27.7%). **Results:** Probable sarcopenia was noted as good-excellent discrimination against across all tools (AUCs 0.764–0.812); confirmed sarcopenia was noted as SARC-CalF superior (AUC = 0.743), followed by SARC-F + EBM (0.697), the Ishii score as moderate (0.667), and SARC-F was limited (0.591; *p* < 0.001 vs. augmented). SARC-CalF optimal cut-offs varied significantly: 4–6 (probable) vs. ≥11 (confirmed). Sex-stratified outcomes had excellent probable detection in both sexes, and this was confirmed to be superior in men. The Ishii score thresholds were 152/244 vs. Asian ≥ 105/120. Obesity required higher cut-offs with high NPVs (77–100%), confirming rule-out utility and SARC-F + EBM performing the best, both in the obesity and sarcopenic obesity subgroups (AUCs 0.742, 0.964). **Conclusions:** Augmented SARC-F scores outperformed the original SARC-F for confirmed sarcopenia in multimorbid Europeans, with SARC-F CalF having the best performance overall. Population-specific (sex/obesity) data-driven thresholds are essential, especially for the Ishii score, as this first Romanian validation reveals limitations of Asian norms in European cohorts, thus advocating for European recalibration.

## 1. Introduction

Sarcopenia is characterized by a progressive decline in muscle strength, function, and mass, leading to an increased risk of falls, disability, hospitalization, and mortality in older adults [1,2,3]. Its prevalence is rising in hospitalized older populations and represents a growing healthcare burden [4].

Obesity is a major risk factor for cardiometabolic multimorbidity and mortality among aging populations, increasing the risk of myocardial infarction, stroke, and type 2 diabetes up to four times [5]. It has increased markedly worldwide over recent decades, with Eastern European countries, including Romania, reporting some of the highest rates in Europe [6,7,8].

Importantly, these two conditions do not always occur in isolation. Sarcopenia and obesity may coexist, as described in the Donini et al. consensus on sarcopenic obesity, where they share partially overlapping pathophysiological mechanisms (including chronic low-grade inflammation, insulin resistance, hormonal dysregulation, and physical inactivity) that synergistically contribute to adverse outcomes such as disability, falls, cardiovascular events, and increased mortality [9]. Sarcopenic obesity (SO) thus emerges as a distinct clinical syndrome defined by elevated adiposity alongside reduced skeletal muscle mass and function [10], conferring a substantially higher overall risk of adverse health outcomes in older adults, compared with sarcopenia or obesity alone [11,12,13]. A recent systematic review by Gao et al. assessing the worldwide prevalence of SO has reported a pooled prevalence of 21% in South America, North America, Asia, Oceania, and Europe [14]. SO also appears to be more prevalent among women and older adults, irrespective of the diagnostic criteria applied [10,15].

The SARC-F questionnaire was the first tool proposed by Malstrom et.al. in 2013 for the screening of sarcopenia [16]. SARC-F is a questionnaire that includes five domains as follows: strength, walking, rising from a chair, climbing stairs, and falls. This screening method has been approved by the EWGSOP2 [1] and has been translated and validated in multiple languages, including Romanian [17]. Despite its high specificity and relatively good diagnostic accuracy, because its sensitivity is low, modified versions of SARC-F have been proposed [18]. These versions include simple anthropometric parameters, such as CalF circumference for SARC-CalF [19], or body mass index (BMI) and age for SARC-F + EBM [20], in order to improve the SARC-F diagnostic power across different populations.

The Ishii score is a screening tool developed and validated in Japan by Ishii et al. to estimate the probability of sarcopenia in older adults. It combines three easily obtainable clinical parameters—age, handgrip strength, and calf circumference—into a sex-specific predictive formula, allowing rapid risk stratification without the need for advanced imaging or complex functional testing [21].

In the original and subsequent validation studies, the Ishii score has shown high diagnostic accuracy, with excellent positive and negative predictive values for identifying individuals with low muscle mass and function when compared with reference standards such as DXA or BIA [21,22,23]. However, most of this research has been conducted in Asian cohorts, often in community-dwelling or outpatient settings, which raises concerns about ethnic and anthropometric differences that may limit the generalizability of its cut-offs and performance to non-Asian, particularly European, hospitalized older populations [22,23,24,25,26,27,28,29,30,31].

Several studies have now examined the performance of sarcopenia screening tools in multi-ethnic cohorts, consistently reporting notable variability in diagnostic accuracy and optimal thresholds across populations [24,26,32]. These findings underscore the need for further research to refine and validate population-specific cut-points that account for ethnic, anthropometric, and body composition differences in order to improve the clinical utility of sarcopenia screening in diverse settings [32,33,34,35,36].

The objective of this study was to compare the diagnostic accuracy of SARC-F, SARC-CalF, SARC-F + EBM, and the Ishii score for identifying EWGSOP2 probable and confirmed sarcopenia in a cohort of Romanian geriatric inpatients, including predefined subgroup analyses by sex and obesity status. We hypothesized that augmented screening tools would outperform the original SARC-F and that the Ishii score would require population-specific thresholds. To our knowledge, this is the first study to provide Romanian data on these instruments, addressing an important gap for their clinical implementation in European geriatric practice.

## 2. Materials and Methods

### 2.1. Study Design

A cross-sectional retrospective analysis was conducted on the data collected from inpatients of the Geriatric ward of the Clinical Municipal Hospital in Cluj-Napoca, Romania, between January 2023 and December 2025. Patients were admitted via a general practitioner referral for geriatric assessment. All patients provided written informed consent upon hospital admission. All procedures were conducted in compliance with the ethical principles outlined in the Declaration of Helsinki and its subsequent revisions. This study was approved by the Ethics Committee of the Municipal Clinical Hospital, Approval No. 2/26.02.2026.

### 2.2. Patient Characteristics and Inclusion Criteria

**Figure 1:**
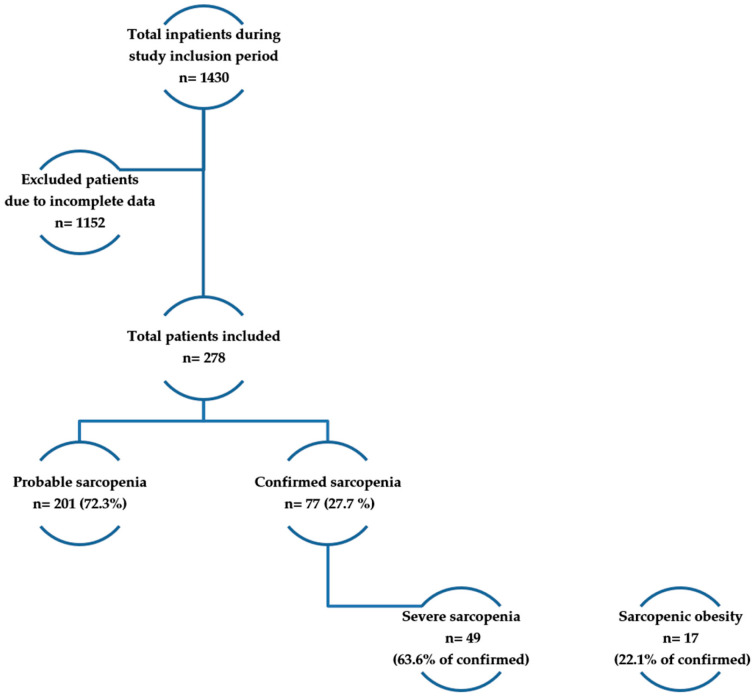
Flowchart of patient inclusion and sarcopenia classification.

All hospitalized patients originated from the Geriatrics Department, as depicted in Figure 1. We included in the analysis patients aged 60 years or more, with the ability to undergo the assessment of body composition with the bioimpedance analysis (BIA) method, in order to obtain appendicular lean mass (ALM) and the ability to perform a 4 m walking speed test.

Patients with incomplete data due to an inability to stand or walk independently, severe language or hearing impairments, cognitive impairment, terminal disease, and patients in whom a sarcopenia status could not be established were excluded. There was no loss due to follow-up, as the study was cross-sectional on retrospective data.

### 2.3. Procedures

The data were extracted from medical reports, including name, personal ID, age, sex, place of origin, education, hospitalisation duration, anthropometric measurements, functionality questionnaires (the ADL Barthel index—Minimum Data Set (MDS), Version 3.0— and the IADL Lawton index), hand grip strength, and answers to sarcopenia screening tools (Table 1).

The ADL index was calculated according to the MDS—Version 3.0, scoring bed mobility, transfer, walking in the room/outside the room, dressing, eating, toilet use, and personal hygiene. The scoring system quantified the level of independence from 0 points (completely independent), 1 point (needing supervision), 2 points (needing limited assistance), 3 points (needing extended assistance), and 4 points (total dependence), and added them up for the total index.

The IADL index was calculated by adding 1 point for needing assistance in each of the following activities: using the telephone, shopping, cooking, cleaning, doing laundry, traveling, taking medication, and money management.

According to the European Working Group on Sarcopenia in Older People (EWGSOP2) first criterion, probable sarcopenia was defined as low muscle strength by measuring hand grip strength (HGS) and the chair stand test (CST).

Confirmed sarcopenia was considered when the first criteria were met, combined with low muscle quantity upon bioelectrical impedance analysis (BIA).

The appendicular lean mass (ALM) was calculated as the sum of all four limbs’ lean mass (kg), and the appendicular lean mass index (ALM index) was calculated by dividing the ALM by height^2^ (kg/m^2^).

The cut-off thresholds recommended by EWGSOP2 were then applied in order to determine the presence of confirmed sarcopenia (ASM < 20 kg for men and <15 kg for women, ASM/height^2^ < 7.0 kg/m^2^ for men and <5.5 kg/m^2^ for women).

Severe sarcopenia was diagnosed in the presence of confirmed sarcopenia with either gait speed < 0.8 m/s, short physical performance battery test (SPPB) < 8 p, or a timed up-and-go test (TUG) > 20 s.

Sarcopenic obesity was defined according to EWGOP2 as the coexistence of sarcopenia and body mass index (BMI) ≥ 30 kg/m^2^.

### 2.4. Measurements

#### 2.4.1. Hand Grip Strength Measurement

Hand grip strength was determined with the Saehan Hydraulic Hand Dynamometer (Item No. SH5001, Serial No. 032465). Patients were seated with their elbows unsupported; the forearm was placed with the elbow in 90 degrees flexion and the wrist in the neutral position. Measurements were made in the dominant and non-dominant hands with the shoulder adducted and neutrally rotated [37].

#### 2.4.2. Lower Body Strength Measurement

The chair stand test measured the time (s) required to stand five times from a seated position on a chair without using the arms. The cut-off values were HGS < 27 kg for men and <16 kg for women, and/or a chair stand test (CST) > 15 s for both sexes.

#### 2.4.3. BMI and Body Composition Assessment

Body composition and weight were assessed using a multifrequency bioelectrical impedance analysis (BIA) system (Visbody-R Explorer 3D Body Scanner, Visbody Intelligent Technology Co., Ltd., Xi’an, China). This device integrates segmental impedance measurements with three-dimensional optical body scanning technology to estimate body composition parameters, including appendicular lean mass (ALM). Measurements were performed with the participants standing barefoot on the device platform, according to the manufacturer’s standardized protocol.

Body height was measured using a medical stadiometer (ASTRA scale with height meter, REF 27310, GIMA S.p.A.–Via Marconi, Gessate (MI), Italy), and the BMI was calculated as weight normalized to height (kg/m^2^).

#### 2.4.4. Physical Performance Assessment

Gait speed was determined by timing the patient while walking at a normal speed for a distance of 4 m, with a 2 m acceleration time in advance.

The short physical performance battery (SPPB) was calculated using three components: the standing balance test, gait speed, and time to rise from a chair five times. For the standing balance, we evaluated the ability to stand for up to 10 s with feet positioned together side-by-side, in semi-tandem and tandem. A score was given depending on the ability to maintain balance in each of these positions. For the other two tests, scores were given based on the time needed to complete the tasks. Each task was scored from 0 to 4, with the scores from the three tests summed to give a total, with a maximum of 12 points [38,39].

The timed up-and-go test (TUG) was used to measure in seconds the time needed for each patient to stand up from a standard chair, walk a distance of 3 m, turn, walk back to the chair, and sit down again. If needed, a walking aid was allowed, but no physical assistance was given [40].

### 2.5. Statistical Analysis

Statistical analyses were performed using jamovi (version 2.5.3; The jamovi project, 2024) with R as the underlying computation engine (R version 4.3; R Core Team, 2023). Continuous variables were summarized as mean ± standard deviation (SD), and categorical variables as counts and percentages. Normality was assessed using the Shapiro–Wilk test. Between-group comparisons for continuous variables were performed using the Student’s *t*-test, and when normal distribution assumptions were not met, the Mann–Whitney U test was used. Categorical variables were compared using the chi-square test; Fisher’s exact test was applied when expected cell counts were small (<5). Receiver operating characteristic (ROC) analyses were conducted to evaluate the discriminative ability of SARC-F, SARC-CalF, SARC-F + EBM, and the Ishii score for EWGSOP2 probable and confirmed sarcopenia. Discrimination was quantified using the area under the ROC curve (AUC). Optimal cut-off values were determined by maximizing the Youden index (sensitivity + specificity − 1), and the values for diagnostic parameters (sensitivity, specificity, positive predictive value, and negative predictive value) were reported as well for selected thresholds. Differences between the AUCs were evaluated using DeLong’s test. Subgroup analyses were performed by sex and obesity status (BMI ≥ 30 kg/m^2^). The *p*-values < 0.05 were considered statistically significant. Effect sizes were calculated. Cohen’s d was used for parametric comparisons (Student’s *t*-test), while rank-biserial correlation (r) was calculated for non-parametric comparisons (Mann–Whitney U test). For categorical variables, the effect size was assessed using Cramér’s V.

AI-tools, such as Perplexity and ChatGPT 5, were used to improve the manuscript’s language and grammar. After using these tools, the authors reviewed and edited the content as needed, and they take full responsibility for the publication’s content.

## 3. Results

### 3.1. Study Population Characteristics

The cohort comprised 278 hospitalized older adults (71.58% female), with probable sarcopenia, identified in 201 participants (72.3%), and confirmed sarcopenia in 77 (27.7%). Obesity had an overall prevalence of 46.76% in our cohort, increasing up to 50.75% among female participants.

#### 3.1.1. Probable Sarcopenia

- Participants with probable sarcopenia were older and exhibited markedly impaired physical function compared to those without (all *p* < 0.001). This included a substantially lower handgrip strength, slower gait speed, reduced SPPB scores, longer TUG time, and fewer 30 s chair stand repetitions. Appendicular lean mass and the ALM index were also reduced (both *p* ≤ 0.003), while screening scores were consistently higher (SARC-F, SARC-CalF, SARC-F + EBM, Ishii; all *p* < 0.001). Sex distribution and urban/rural residence were comparable between groups (both *p* > 0.05), but hospital length of stay was longer among those with probable sarcopenia (*p* < 0.001).

#### 3.1.2. Confirmed Sarcopenia

Confirmed sarcopenia cases had lower anthropometric measures (weight, height, BMI; all *p* < 0.001) and reduced muscle mass indices (ALM, ALM index; both *p* < 0.001) compared to non-sarcopenic cases. Muscle strength was markedly lower (handgrip strength, both hands: *p* < 0.001), gait speed was slower (0.68 ± 0.30 m/s vs. 0.82 ± 0.33 m/s; *p* = 0.001), and SPPB scores were slightly reduced (6.86 ± 3.13 vs. 7.87 ± 3.03; *p* = 0.017). Composite screening scores were elevated (SARC-CalF, SARC-F + EBM, Ishii; all *p* ≤ 0.002), while SARC-F showed only a marginal difference (*p* = 0.021).

Severe sarcopenia, obesity, and sarcopenic obesity had a higher prevalence among women, while sarcopenic obesity had a prevalence of 22.1% out of all confirmed cases, though hospital stay did not differ (*p* = 0.888). Baseline characteristics are represented in Table 2.

In men, differences between sarcopenic and non-sarcopenic patients were associated with large effect sizes for handgrip strength (d = 0.78–0.91) and gait speed (d = 0.98), while functional performance measures showed small to moderate effects. Furthermore, severe sarcopenia was associated with very large effect sizes for handgrip strength (d = 1.08–1.15) and gait speed (d = 1.15), indicating a pronounced functional impairment and strong separation between groups.

Physical performance parameters, according to confirmed sarcopenia status, stratified by sex, are represented in Table 3, and physical performance parameters according to severe sarcopenia status, stratified by sex, are represented in Table 4.

### 3.2. Diagnostic Performance of Screening Tools in the Overall Cohort

ROC curve analysis was performed to compare the diagnostic performance of the four screening tools for sarcopenia in this Romanian cohort. For probable sarcopenia, all instruments yielded acceptable to good discrimination (AUCs 0.764–0.812), and no statistically significant differences were detected between AUCs, according to DeLong’s overall test (*p* = 0.523). The results of this section are summarized in Table 5.

In contrast, the ability to discriminate confirmed sarcopenia varied more clearly across instruments. SARC-CalF achieved the highest accuracy (AUC = 0.743) at 11 points, followed by SARC-F + EBM (AUC = 0.697) at 12 points and the Ishii score (AUC = 0.667) at 229 points, while the original SARC-F showed only limited discrimination (AUC = 0.591) at 4 points.

For the Ishii score, the best Youden performance occurred at 229.6–229.8 overall for confirmed sarcopenia (sensitivity 40.3%, specificity 83.6%; AUC = 0.667), reflecting population-specific recalibration needs. Optimal cut-offs varied markedly by outcome and sex in our Romanian cohort: ~116/152 (men probable/confirmed), ~206/244 (women probable/confirmed).

DeLong’s overall test confirmed significant differences among the ROC curves for confirmed sarcopenia (*p* < 0.001). Pairwise comparisons indicated that SARC-F performed significantly worse than SARC-F CalF (ΔAUC = −0.152, *p* < 0.001) and SARC-F + EBM (ΔAUC = −0.106, *p* < 0.001), whereas differences among SARC-F CalF, SARC-F + EBM, and the Ishii score were not statistically significant (all *p* > 0.05).

**Figure 2:**
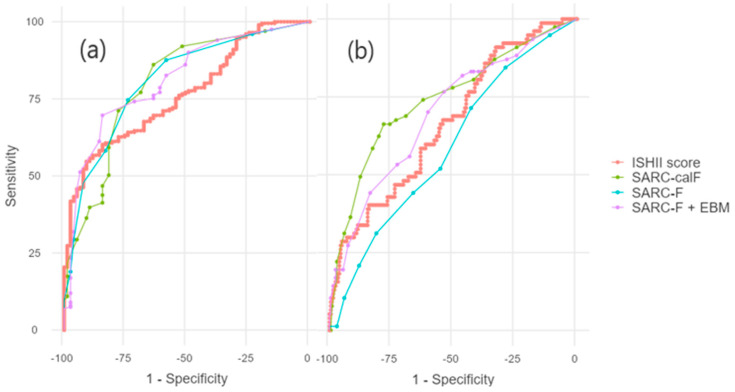
Receiver operating characteristic (ROC) curves showing the diagnostic performance of sarcopenia screening scores: (**a**) Discrimination of probable sarcopenia and (**b**) discrimination of confirmed sarcopenia. Curves are shown for the Ishii score, SARC-F (strength, assistance walking, rise from a chair, climb stairs, falls), SARC-CalF (SARC-F with added CalF circumference) and SARC-F + EBM (SARC-F with body mass index and age).

Detailed operating characteristics for each cut-off, including sensitivity, specificity, and the Youden index, are summarized in Table 6, and combined ROC curves of the four screening tools for probable and confirmed sarcopenia are represented in Figure 2.

### 3.3. Sex-Specific Diagnostic Performance

#### 3.3.1. Probable Sarcopenia

All scores demonstrated good to excellent discrimination in both sexes. In men, the Ishii score (AUC = 0.877), SARC-F (AUC = 0.872), and SARC-F + EBM (AUC = 0.868) achieved excellent performance, outperforming SARC-CalF (AUC = 0.803). In women, the Ishii score led (AUC = 0.866), followed by SARC-CalF (AUC = 0.801), SARC-F + EBM (AUC = 0.788), and SARC-F (AUC = 0.782). Optimal cut-points maintained high sensitivity and specificity for all screening tools across sexes (men: 70.91–83.64% sensitivity, 79.17–91.67% specificity; women: 67.12–76.03% sensitivity, 66.04–86.79% specificity), with the mention of the Ishii score having significantly higher thresholds for women (206) in order to obtain this diagnostic performance.

#### 3.3.2. Confirmed Sarcopenia

Performance varied more markedly. In men, augmented scores excelled: SARC-F + EBM and SARC-CalF (AUCs 0.824, 0.821), followed by the Ishii score (0.773) and SARC-F (0.703). In women, SARC-CalF performed best (AUC = 0.716), while SARC-F showed poor discrimination (0.546); SARC-F + EBM and the Ishii score were modest (0.650, respectively 0.674). Across sexes, NPVs were consistently high (77–100%), indicating better utility of the screening scores for ruling out sarcopenia than confirming it.

### 3.4. Diagnostic Performance by Obesity Status

#### 3.4.1. Probable Sarcopenia

Normal-weight individuals showed superior overall discrimination of probable sarcopenia compared to participants with obesity. SARC-F excelled (AUC = 0.859), followed by SARC-CalF (AUC = 0.821), SARC-F + EBM (AUC = 0.806), and the Ishii score (AUC = 0.786). Individuals with obesity exhibited modestly lower AUCs (0.783–0.823), requiring slightly higher thresholds for almost all screening tools (SARC-F/SARC-CalF ≥ 6; SARC-F + EBM ≥ 12; Ishii ~208) to maintain specificity.

#### 3.4.2. Confirmed Sarcopenia

Participants with obesity demonstrated moderate, consistent performance across scores (AUCs 0.701–0.742). SARC-F + EBM performed best, with a cut-off of 13. Normal-weight participants showed greater heterogeneity of screening scores performance compared to the probable sarcopenia analysis: SARC-CalF performed best (AUC = 0.717), followed by the Ishii score (0.703), SARC-F + EBM (0.671), while SARC-F performance was limited (0.610).

#### 3.4.3. Sarcopenic Obesity Subgroup

In sarcopenic obesity (SO) cases, performance was excellent (AUC 0.833–0.964), with higher cut-offs (SARC-F/SARC-CalF = 6 and SARC-F + EBM ≥ 14; Ishii ≥ 220), yielding 100% specificity/PPV with variable sensitivity across scores; however, the results were limited by the small subgroup size.

### 3.5. Main Diagnostic Findings

Screening tools demonstrated comparable good-to-excellent performance for probable sarcopenia across cohort, sex, and obesity strata (AUCs 0.764–0.812), with some differences regarding thresholds (SARC-CalF 4–6 for probable sarcopenia and 11 for confirmed sarcopenia; Ishii score ≥ 116/206 for probable sarcopenia (men/woman) and ≥152/244 for confirmed sarcopenia (men/woman).

Confirmed sarcopenia showed marked heterogeneity with SARC-CalF consistently being superior (highest AUCs overall), followed by SARC-F + EBM (best performance in the obesity subgroup), the Ishii score as moderate (third-ranked), and SARC-F as limited/inferior (DeLong *p* < 0.001).

Sex patterns: There was excellent probable sarcopenia detection in both sexes, while confirmed sarcopenia was markedly better in men (all AUCs > 0.70) vs. moderate-poor in women (SARC-F notably weak).

Obesity effects: Masked signals requiring higher thresholds with sarcopenic obesity had a near-perfect specificity (100% PPV).

High NPVs (77–100%) across the subgroups confirm rule-out superiority for clinical screening.

The results of this section are represented in Table 7, comparing the screening scores performance across all the subgroups with the total population.

## 4. Discussion

### 4.1. Physical Performance in Sarcopenic Inpatients

Our findings suggest that confirmed sarcopenia was associated with substantial physical performance deficits in geriatric inpatients, particularly among men, where handgrip strength and gait speed showed large effect sizes, consistent with EWGSOP2 criteria emphasizing muscle strength as the primary diagnostic anchor. These impairments were even more pronounced in severe sarcopenia, highlighting a gradient of functional decline that underscores the clinical relevance of severity staging in hospitalized older adults. Notably, women exhibited smaller effect sizes, potentially reflecting sex-specific muscle quality differences or lower baseline strength levels in Eastern European cohorts [41].

Superior discrimination by handgrip and gait speed aligns with prior validations of EWGSOP2 in acute care settings, where these parameters best capture sarcopenia’s functional consequences. However, the modest effects on chair stand tests and SPPB in women suggest these tools may have limited sensitivity for milder sarcopenia phenotypes, supporting calls for adjusted cut-offs.

### 4.2. SARC-F Performance Compared to Augmented Sores

Augmented screening scores emerged as superior to the original SARC-F in our cohort, consistent with previous evidence [19,20,25,33]. SARC-CalF and SARC-F + EBM consistently achieved higher AUCs for confirmed sarcopenia, with DeLong pairwise comparisons confirming their statistical advantage over SARC-F. The limited discrimination of SARC-F, particularly among women, mirrors previous reports of its suboptimal sensitivity and indicates that exclusive reliance on the original questionnaire may lead to under-detection of clinically relevant sarcopenia cases [19,20].

In consequence, adding anthropometric measurements such as CC and BMI improves the sensitivity of the score by providing objective indicators of low muscle mass, complementing the subjective assessment of muscle function.

### 4.3. SARC-CalF Performance According to Outcome Definition and Sex

The divergent optimal cut-offs for SARC-CalF between probable (4–6 points) and confirmed sarcopenia (≥11 points) highlight the tool’s sensitivity to outcome definition. Lower thresholds maximized case-finding for probable sarcopenia (low muscle strength/performance), while higher cut-offs better discriminated against confirmed cases requiring additional muscle mass deficits. This pattern differs from the conventional ≥11-point threshold proposed in other cohorts for general sarcopenia risk [19], suggesting population and criteria-specific optimization improves diagnostic accuracy in European hospitalized patients.

These findings support data-driven threshold selection over fixed literature values when implementing screening protocols and align with recent EWGSOP2-based validation, recommending reduced thresholds (SARC-F ≥ 2) to enhance probable sarcopenia detection [42].

Our results also demonstrate clear sex-specific optimal cut-offs that are consistent with physiological differences in muscle mass and strength. In this hospitalized Romanian cohort, these variations highlight limitations of universal thresholds, advocating for sex-stratified guidelines—particularly in multimorbid older adults where diagnostic precision is critical (SARC-F CalF ≥ 4 men vs. ≥6 women around the same AUC = 0.80, for probable sarcopenia).

### 4.4. Ishii Score Performance and Thresholds for the European Population

Interestingly, Ishii score optimal cut-offs in our Romanian cohort (≥116/206 for probable sarcopenia and ≥152/244 for confirmed sarcopenia) substantially exceeded original Japanese thresholds (≥105/120) [21], with female values doubling. In a 2023 China-based study by Luo et.al. [26], a slightly different threshold for the Ishii score (≥114/120) has also been proposed in order to improve its performance for multi-ethnic adults; however, the analysis was limited to severe sarcopenic adults over 50 years of age from the West China Health and Aging Trend (WCHAT) study.

This significant difference in cut-off values could be attributable to variations in anthropometric parameters and a higher prevalence of obesity in Eastern European countries, with a recent global report of adult overweight and obesity prevalence confirming an increase of up to 40% in obesity status among East Europeans compared to South Asians [6,8].

Furthermore, the need for higher thresholds may stem from substantial differences in muscle strength across regions and ethnicities, rendering a universal threshold suboptimal [41,43].

In terms of performance, despite good probable sarcopenia detection (AUCs 0.877 men, 0.866 women), confirmed accuracy dropped to moderate in our cohort (AUC = 0.667, third-ranked vs. augmented SARC-F), being limited by grip strength incorporation bias, Asian community validation context, and BMI/body composition differences [44].

In addition to the threshold differences, its overall performance in detecting sarcopenia was much weaker on this cohort than that of the original validation studies (Ishii et al., AUC = 0.939 [21], Erdogan et al., AUC = 0.961 [36], Li et al., AUC = 0.78 [22], Guo et al., AUC = 0.81 [25], and Peng et al., AUC = 0.846 in men and 0.824 in women [27]). These findings underscore the need for population-specific ROC recalibration before adopting Asian-derived thresholds in the European population, as its direct applicability remains limited outside of Asian settings.

### 4.5. Screening Scores Performance in Patients with Obesity

Obesity systematically elevated optimal cut-offs, reflecting attenuated signals from excess adiposity, with SARC-F + EBM having, overall, the best performance in this subgroup at a threshold of 12–13. This result is consistent with previous studies [20,25] suggesting SARC-F + EBM utility in screening, and it supports the versatility of this screening tool across different populations and weight variations.

### 4.6. Study Limitations

Several methodological limitations warrant acknowledgment. First, we assessed muscle mass via bioelectrical impedance analysis (BIA) rather than dual-energy X-ray absorptiometry (DXA), which is the reference standard for clinical and research applications per EWGSOP2 [45]. BIA tends to overestimate skeletal muscle mass relative to DXA, likely underestimating sarcopenia prevalence in our cohort [46].

Established imaging alternatives like computed tomography (CT) and magnetic resonance imaging (MRI) offer complementary strengths despite their constraints. According to Derstine et al. [47], CT provides a precise skeletal muscle cross-sectional area measurement at L3/T12 using a −29 to 150 Hounsfield Units (HU) threshold, yielding contrast-independent reference values. Chianca et al. position MRI, via Dixon sequences, as superior for radiation-free fat quantification and muscle quality, yet note its expense and limited availability [48]. Integrating CT or MRI could enhance our BIA-based findings, potentially improving diagnostic specificity in geriatric cohorts despite accessibility barriers [47,48,49].

Moreover, the evolution of diagnostic imaging has introduced Point-of-Care (POCUS) tools that overcome the logistical limitations of DXA and BIA. In particular, muscle ultrasound is emerging as a non-invasive, radiation-free, and highly repeatable method, capable of providing immediate quantitative and qualitative data on muscle mass—such as muscle thickness (MT), echogenicity (an indicator of fibro-adipose infiltration), and the pennation angle—directly at the patient’s bedside, as reported by Fraccalini et al. [50]. The results of the present study could be further refined by integrating these advanced morphological parameters, through an assessment that goes beyond simple anthropometric measurements.

Second, SO cases were identified using EWGSOP2 criteria, which yielded a small subgroup size. Applying the more inclusive European Society for Clinical Nutrition and Metabolism and European Association for the Study of Obesity (ESPEN-EASO) consensus might have identified more SO cases and improved screening performance estimates [51]. Third, as an observational, single-center study on Romanian inpatients, our findings may have limited generalizability to other populations with different body compositions, nutritional status, or morbidity profiles. Future multicenter, prospective validation studies are warranted.

## 5. Conclusions

In this hospitalized Romanian cohort, augmented SARC-F scores (SARC-CalF, SARC-F + EBM) demonstrated superior discrimination for confirmed sarcopenia versus original SARC-F and the Ishii score, while all tools performed comparably well for probable cases. Population-specific thresholds proved essential: SARC-CalF optimal cut-offs varied from 4–6 (probable) to ≥11 (confirmed). The Ishii score required a threshold of 152/244 (vs. Asian ≥ 105/120), doubling this for European women.

Sex-stratified patterns revealed excellent male performance across outcomes, moderate-to-poor female confirmed detection (SARC-F notably weak), and obesity-masked signals, necessitating higher thresholds. High NPVs (77–100%) confirmed rule-out utility across subgroups.

These first Romanian data highlight important limitations of Asian-validated sarcopenia screening norms in multimorbid European inpatients, advocating data-driven, sex- and obesity-adjusted thresholds—with SARC-CalF as the optimal triage tool—and recommending population-specific recalibration.

## Figures and Tables

**Table 1:** Sarcopenia screening tools.

Score	Components	Scoring	Cut-Off for Confirmed Sarcopenia
^1^ SARC-F	Strength Assistance walking Rise from a chair Climb stairs Falls	Each 0–2 0 = none 1 = some 2 = a lot/unable Total: 0–10	≥4
^2^ SARC-CalF	SARC-F + Calf circumference	SARC-F (0–10) +	≥11
		CC > 33/34 cm: 0	
		CC ≤ 33/34 cm: 10 Total: 0–20	
^3^ SARC-F + EBM	SARC-F + Elderly + BMI	SARC-F (0–10) +	≥12
		Age < 75 years: 0	
		Age ≥ 75 years: 10	
		BMI > 21 kg/m^2^: 0	
		BMI ≤ 21 kg/m^2^: 10 Total: 0–30	
^4^ Ishii score	Age Grip strength CC	M: 0.62 (age-64)–3.09 (grip-50)–4.64 (CC-42)	M ≥ 105
		F: 0.80 (age-64)–5.09 (grip-34)–3.28 (CC-42)	F ≥ 120

Abbreviations: ^1^ SARC-F questionnaire—strength, assistance walking, rise from a chair, climb stairs, falls; ^2^ SARC-CalF—SARC-F with added CalF circumference; ^3^ SARC-F + EBM—SARC-F with body mass index and age; ^4^ Ishii score—formula as presented.

**Table 2:** Baseline characteristics of the study population according to sex.

Variable	Overall (*n* = 278)	Females (*n* = 199)	Males (*n* = 79)	*p*-Value, Effect Size
Age (years)	77.69 ± 7.47	77.21 ± 7.28	78.93 ± 7.85	0.078 *, 0.135
Rural residence, *n* (%)	106 (38.12)	80 (40.20)	26 (32.91)	0.259 ^#^, 0.067
Weight (kg)	76.51 ± 16.96	73.96 ± 16.81	82.94 ± 15.66	<0.001 *, 0.337
Height (cm)	159.19 ± 8.94	155.40 ± 6.08	168.74 ± 7.80	<0.001 *, 0.833
^1^ BMI (kg/m^2^)	30.19 ± 6.26	30.62 ± 6.64	29.11 ± 5.07	0.042, 0.242
^2^ SARC-F	3.57 ± 2.44	3.79 ± 2.47	3.01 ± 2.31	0.017 *, 0.182
^3^ SARC-CalF	6.95 ± 5.52	7.01 ± 5.40	6.81 ± 5.83	0.486 *, 0.053
^4^ SARC-F + EBM	10.69 ± 6.95	10.77 ± 6.93	10.48 ± 7.03	0.463 *, 0.056
^5^ Ishii score	185.87 ± 54.64	210.77 ± 37.38	123.16 ± 38.60	<0.001 *, 0.895
^6^ ADL score	2.68 ± 4.43	2.83 ± 4.70	2.32 ± 3.66	0.500 *, 0.047
^7^ IADL score	3.46 ± 2.89	3.51 ± 2.87	3.34 ± 2.95	0.626 *, 0.037
Calf circumference (cm)	35.71 ± 4.80	35.84 ± 4.86	35.41 ± 4.64	0.801 *, 0.019
^8^ ALM (kg)	18.59 ± 4.61	16.69 ± 2.91	23.38 ± 4.63	<0.001, 1.919
^9^ ALM index (kg/m^2^)	7.24 ± 1.16	6.88 ± 0.92	8.16 ± 1.22	<0.001, 1.264
Probable Sarcopenia, *n* (%)	201 (72.30)	146 (73.36)	55 (69.62)	0.529 ^#^, 0.037
Confirmed Sarcopenia, *n* (%)	77 (27.69)	59 (29.65)	18 (22.78)	0.249 ^#^, 0.069
Severe sarcopenia, *n* (%)	49 (17.63)	34 (17.08)	15 (18.98)	0.707 ^#^, 0.022
Obesity, *n* (%)	130 (46.76)	101 (50.75)	29 (36.71)	0.034 ^#^, 0.126
Sarcopenic obesity, *n* (%)	17 (6.11)	15 (7.53)	2 (2.53)	0.165 ^##^

Note: Data are presented as mean ± standard deviation or *n* (%). Abbreviations: ^1^ BMI—body mass index, ^2^ SARC-F questionnaire—strength, assistance walking, rise from a chair, climb stairs, falls; ^3^ SARC-CalF—SARC-F with added CalF circumference; ^4^ SARC-F + EBM—SARC-F with body mass index and age; ^5^ Ishii score—formula as presented in Table 1; ^6^ ADL score—activities of daily living; ^7^ IADL—independent activities of daily living; ^8^ ALM—appendicular lean mass; ^9^ ALM index—appendicular lean mass index. * Mann–Whitney U test; no symbol—Student’s *t*-test. ^#^ Chi-square test; ^##^ Fisher’s exact test. Effect size was reported as Cohen’s d for parametric comparisons (Student’s *t*-test) and rank-biserial correlation (r) for non-parametric comparisons (Mann–Whitney U test). Cramér’s V was used for categorical variables where applicable.

**Table 3:** Physical performance parameters according to confirmed sarcopenia, stratified by sex.

	Male			Female		
Physical Performance Parameters	Without Sarcopenia (*n* = 61)	Sarcopenia (*n* = 18)	*p*-Value, Effect Size	Without Sarcopenia (*n* = 140)	Sarcopenia (*n* = 59)	*p*-Value, Effect Size
Handgrip strength right (kg)	29.52 ± 9.20	22.50 ± 7.79	0.004, 0.788	18.15 ± 5.88	14.78 ± 5.68	0.001 *, 0.292
Handgrip strength left (kg)	29.04 ± 9.11	21.03 ± 7.44	0.001, 0.913	16.78 ± 6.42	12.96 ± 5.60	<0.001 *, 0.358
Gait speed (m/s)	0.97 ± 0.36	0.64 ± 0.24	<0.001, 0.987	0.76 ± 0.30	0.69 ± 0.31	0.129, 0.236
Five-time chair stand test (s)	17.79 ± 13.71	27.83 ± 19.90	0.058 *, 0.297	21.03 ± 14.04	23.16 ± 16.67	0.717 *, 0.032
Chair rise test (30 s reps)	10.43 ± 4.73	7.44 ± 5.39	0.026, 0.610	8.96 ± 4.28	8.39 ± 4.38	0.612 *, 0.045
^1^ SPPB score	8.62 ± 3.15	6.50 ± 3.35	0.015 *, 0.352	7.54 ± 2.93	6.97 ± 3.08	0.236 *, 0.106
Timed Up and Go (s)	15.69 ± 12.76	18.90 ± 10.29	0.022 *, 0.357	17.04 ± 12.49	20.03 ± 15.82	0.250 *, 0.103

Note: Data are presented as mean (SD). Sarcopenia = confirmed sarcopenia. Abbreviations: ^1^ SPPB—short physical performance battery. * Mann–Whitney U test; no symbol—Student’s *t*-test. Effect size was reported as Cohen’s d for parametric comparisons (Student’s *t*-test) and rank-biserial correlation (r) for non-parametric comparisons (Mann–Whitney U test).

**Table 4:** Physical performance parameters according to severe sarcopenia status, stratified by sex.

	Male			Female		
Physical Performance Parameters	Without Severe Sarcopenia (*n* = 64)	Severe Sarcopenia (*n* = 15)	*p*-Value, Effect Size	Without Severe Sarcopenia (*n* = 165)	Severe Sarcopenia (*n* = 34)	*p*-Value, Effect Size
Handgrip strength right (kg)	29.70 ± 9.08	20.33 ± 6.22	<0.001, 1.085	17.93 ± 5.87	13.35 ± 5.23	<0.001 *, 0.438
Handgrip strength left (kg)	29.09 ± 8.96	19.20 ± 6.38	<0.001, 1.157	16.54 ± 6.44	11.31 ± 4.21	<0.001 *, 0.528
Gait speed (m/s)	0.96 ± 0.35	0.58 ± 0.23	<0.001, 1.157	0.79 ± 0.29	0.47 ± 0.22	<0.001 *, 0.638
Five-time chair stand test (s)	17.38 ± 13.52	31.60 ± 19.72	0.017 *, 0.531	19.95 ± 13.23	29.98 ± 19.18	0.001 *, 0.357
Chair rise test (30 s reps)	10.66 ± 4.77	5.87 ± 4.22	<0.001 *, 0.563	9.26 ± 4.12	6.50 ± 4.51	<0.001 *, 0.367
^1^ SPPB score	8.77 ± 3.14	5.47 ± 2.59	<0.001 *, 0.580	7.86 ± 2.86	4.97 ± 2.34	<0.001 *, 0.566
Timed Up and Go (s)	15.41 ± 12.52	20.75 ± 10.31	0.002 *, 0.525	16.10 ± 11.75	26.76 ± 18.05	<0.001 *, 0.542

Note: Data are presented as mean (SD). Sarcopenia = confirmed sarcopenia. Abbreviations: ^1^ SPPB—short physical performance battery. * Mann–Whitney U test; no symbol—Student’s *t*-test. Effect size was reported as Cohen’s d for parametric comparisons (Student’s *t*-test) and rank-biserial correlation (r) for non-parametric comparisons (Mann–Whitney U test).

**Table 5:** Diagnostic performance of all screening tools for probable sarcopenia on overall cohort.

Score	Optimal Cut-Off (≥4)	Sensitivity (%)	Specificity (%)	AUC	Youden Index
^1^ SARC-F	4	74.63	74.03	0.808	0.49
^2^ SARC-CalF	4	86.07	63.64	0.800	0.49
^3^ SARC-F + EBM	12	69.65	84.42	0.812	0.54
^4^ Ishii score	206	54.23	90.91	0.764	0.45

Abbreviations: ^1^ SARC-F questionnaire—strength, assistance walking, rise from a chair, climb stairs, falls; ^2^ SARC-CalF—SARC-F with added CalF circumference; ^3^ SARC-F + EBM—SARC-F with body mass index and age; ^4^ Ishii score—formula as presented.

**Table 6:** Diagnostic performance of all screening tools for confirmed sarcopenia on overall cohort.

Score	Optimal Cut-Off	Sensitivity (%)	Specificity (%)	AUC	Youden Index
^1^ SARC-F	4	71.43	42.79	0.591	0.14
^2^ SARC-CalF	11	66.23	78.11	0.743	0.44
^3^ SARC-F + EBM	12 *	76.62	53.73	0.697	0.30
^4^ Ishii score	229	40.26	83.08	0.667	0.23

Abbreviations: ^1^ SARC-F questionnaire—strength, assistance walking, rise from a chair, climb stairs, falls; ^2^ SARC-CalF—SARC-F with added CalF circumference; ^3^ SARC-F + EBM—SARC-F with body mass index and age; ^4^ Ishii score—formula as presented. * Cut-off ≥ 13 yields a similar Youden index with higher specificity (60.2%) and lower sensitivity (70.1%).

**Table 7:** Diagnostic performance and optimal cut-off values of sarcopenia screening scores across subgroups.

**A. Probable Sarcopenia**			
**Overall Study Group**	**Men**	**Women**	**Patients with Obesity**
^1^ SARC-F: ≥4 | AUC ≈ 0.808 ^2^ SARC-CalF: ≥4 | AUC ≈ 0.800 ^3^ SARC-F + EBM: ≥12 | AUC ≈ 0.812 ^4^ Ishii score: ≥206 | AUC ≈ 0.764	SARC-F: ≥4 | AUC = 0.872	SARC-F: ≥4 | AUC = 0.782	SARC-F: ≥6 | AUC = 0.792
	SARC-CalF: ≥4 | AUC = 0.803	SARC-CalF: ≥6 | AUC = 0.801	SARC-CalF: ≥6 | AUC = 0.783
	SARC-F + EBM: ≥8 | AUC = 0.868 Ishii score: ≥116 | AUC = 0.877	SARC-F + EBM: ≥12 | AUC = 0.788 Ishii score: ≥206 | AUC = 0.866	SARC-F + EBM: ≥12 | AUC = 0.823 Ishii score: ≥208 | AUC = 0.761
**B. Confirmed Sarcopenia**			
**Overall Study Group**	**Men**	**Women**	**Patients with Obesity**
^1^ SARC-F: ≥4 | AUC ≈ 0.591 ^2^ SARC-CalF: ≥11 | AUC ≈ 0.743 ^3^ SARC-F + EBM: ≥12 | AUC ≈ 0.697 ^4^ Ishii score: ≥229 | AUC ≈ 0.667	SARC-F: ≥4 | AUC = 0.703	SARC-F: ≥7 | AUC = 0.546	SARC-F: ≥6 | AUC = 0.701
	SARC-CalF: ≥11 | AUC = 0.821	SARC-CalF: ≥11 | AUC = 0.715	SARC-CalF: ≥7 | AUC = 0.711
	SARC-F + EBM: ≥12 | AUC = 0.824 Ishii score: ≥152 | AUC = 0.773	SARC-F + EBM: ≥12 | AUC = 0.649 Ishii score: ≥244 | AUC = 0.673	SARC-F + EBM: ≥13 | AUC = 0.742 Ishii score: ≥255 | AUC = 0.726

* Abbreviations: ^1^ SARC-F questionnaire—strength, assistance walking, rise from a chair, climb stairs, falls; ^2^ SARC-CalF—SARC-F with added CalF circumference; ^3^ SARC-F + EBM—SARC-F with body mass index and age; ^4^ Ishii score—formula as presented.

## Data Availability

The datasets generated and/or analyzed during the current study are not publicly available due to institutional and ethical regulations but are available from the corresponding author upon reasonable request and with permission of the Ethics Committee.

## Abbreviations

The following abbreviations are used in this manuscript:

SARC-F	Strength, Assistance walking, Rise from a chair, Climb stairs, Falls
SARC-CalF	SARC-F with added calf circumference
SARC-F + EBM	SARC-F with body mass index and age
ADL score	Activities of daily living
IADL	Independent activities of daily living
ALM	Appendicular lean mass
ALM index	Appendicular lean mass index
HGS	Handgrip strength
SPPB	Short physical performance battery
SO	Sarcopenic obesity
WCHAT	West China Health and Aging Trend
ESPEN	European Society for Clinical Nutrition and Metabolism
EASO	European Association for the Study of Obesity
